# A novel peaked cap technique for assisting colonic endoscopic submucosal dissection

**DOI:** 10.1055/a-2738-6966

**Published:** 2025-12-15

**Authors:** Qian Yang, Jia Xu, Jing Cao, Xiaowei Tang

**Affiliations:** 1 556508 Department of Gastroenterology, The Affiliated Hospital of Southwest Medical University, Luzhou, China

The use of a transparent cap with a small-caliber tip has been reported to enhance visual field clarity during endoscopic submucosal dissection (ESD 1 2 ). Previous studies have described the role of such caps in the ESD procedure 3 . Herein, we present a self-designed “peaked cap” featuring an additional peaked cap-style extension ( Video 1 ).

Demonstration of how the “peaked cap” enhances submucosal visualization and optimizes surgical field exposure during ESD, thereby facilitating precise lesion dissection.Video 1

A 77-year-old woman underwent total colonoscopy for abdominal discomfort, revealing a laterally spreading tumor measuring approximately 3 cm × 4 cm in the transverse colon (50 cm from the anal verge). After obtaining informed consent, ESD was performed using a colonoscope equipped with the “peaked cap”. This device was adhered to the transparent cap using a nail art adhesive method ( Fig. 1 **a** and **b** ). The mucosa and submucosa were incised along the marked area using a dual knife in endocut Q mode (Effect 3, ERBE generator; Fig. 1 **c** ). The “peaked cap” facilitated insertion of the dual knife into the submucosal layer for dissection. Its structural design enables active retraction of the incised mucosal flap, maintaining optimal surgical visibility ( Fig. 1 **d** and Fig. 2 ). During the procedure, submucosal dissection was performed smoothly ( Fig. 1 **e** ). Hemostasis was achieved with a coagulating forceps in soft-coagulation mode (Effect 4, 80 W). The post-resection defect was closed with multiple hemostatic clips. Whole resection was successfully completed without adverse events, and the specimen was sent for histopathological examination, revealing a tubulovillous adenoma without vascular or lymphatic invasion ( Fig. 1 **f** and Fig. 3 ). The patient was discharged on postoperative day 3 without complications and remained asymptomatic during 3-month follow-up endoscopy.

**Fig. 1:**
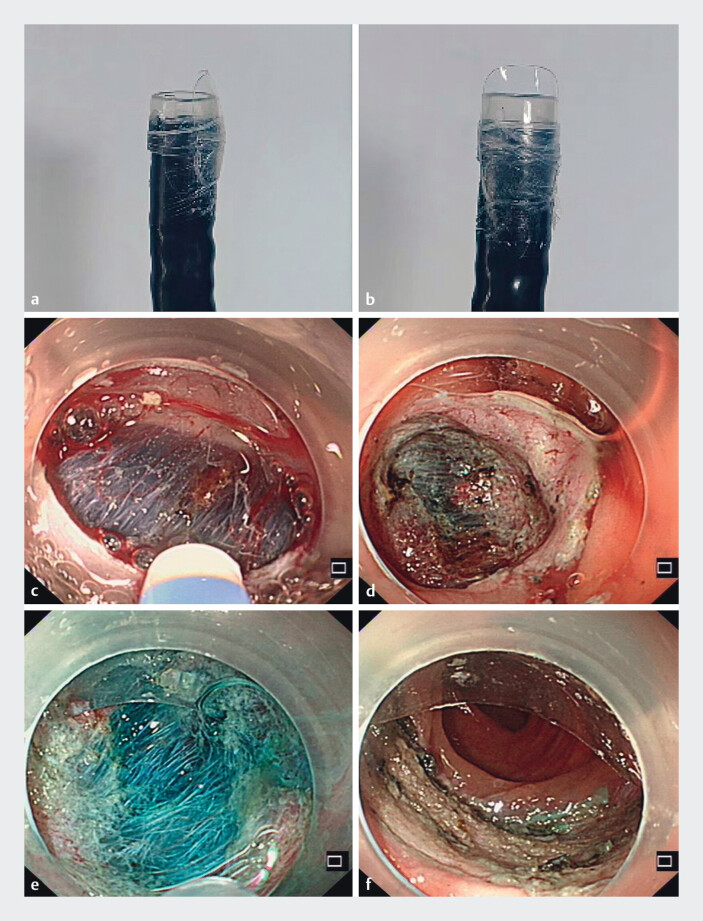
**a** The peaked cap secured with a nail art adhesive technique in this report: lateral view. **b** The peaked cap secured with a nail art adhesive technique in this report: anterior view. **c** The mucosa and submucosa were incised using a dual knife. **d** The visor-style transparent cap deflected the mucosal flap, maintaining optimal submucosal visualization. **e** Surgical field during colonic ESD. **f** Post-resection wound showing hemostasis without oozing.

**Fig. 2:**
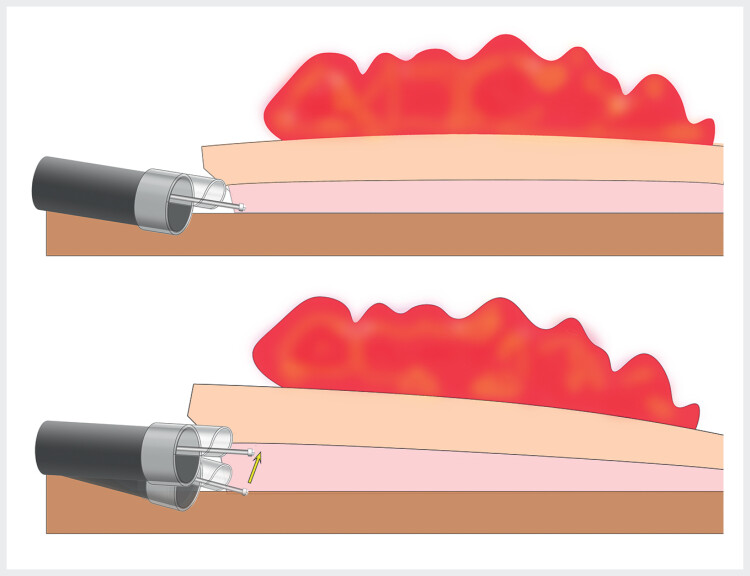
Schematic shows that the “peaked cap” retracts the incised mucosal flap, enhancing surgical field visualization to facilitate precise submucosal dissection.

**Fig. 3:**
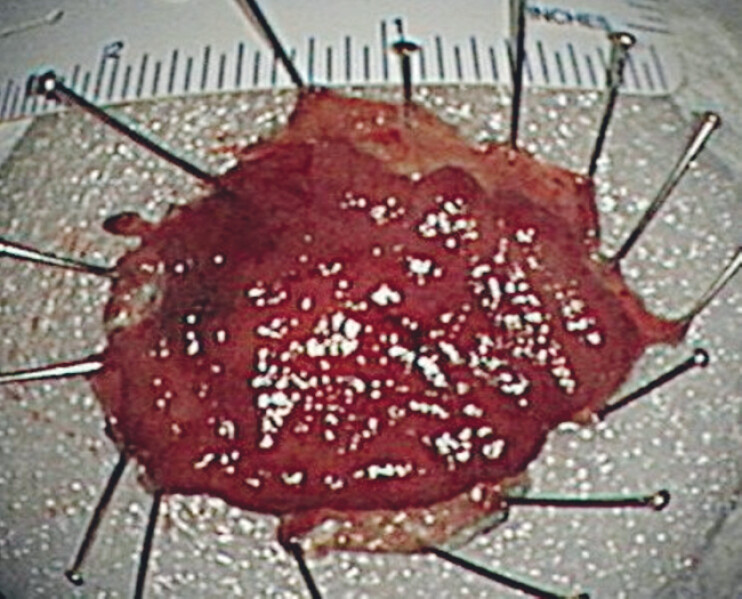
The en bloc resected specimen.

The “peaked cap” technique offers dual clinical–economic merits. The first is that its structural design provides continuous retraction of mucosal flaps, maintaining optimal submucosal visualization that is critical for precise dissection. The second is that its cost-efficiency significantly alleviates financial burdens for patients, particularly valuable in resource-constrained settings where balancing therapeutic efficacy and expenditure is paramount.

Endoscopy_UCTN_Code_TTT_1AQ_2AD_3AD

## Notes

Endoscopy E-Videos https://eref.thieme.de/e-videos
*E-Videos* is an open access online section of the journal *Endoscopy* , reporting on interesting cases and new techniques in gastroenterological endoscopy. All papers include a high-quality video and are published with a Creative Commons CC-BY license. Endoscopy E-Videos qualify for HINARI discounts and waivers and eligibility is automatically checked during the submission process. We grant 100% waivers to articles whose corresponding authors are based in Group A countries and 50% waivers to those who are based in Group B countries as classified by Research4Life (see: https://www.research4life.org/access/eligibility/ ). This section has its own submission website at https://mc.manuscriptcentral.com/e-videos .
